# Biotransformation of Indigo Pigment by Indigenously Isolated *Pseudomonas* sp. HAV-1 and Assessment of Its Antioxidant Property

**DOI:** 10.1155/2014/109249

**Published:** 2014-11-17

**Authors:** Aditi Dua, Kishor Chauhan, Hilor Pathak

**Affiliations:** ^1^Department of Microbiology, P. D. Patel Institute of Applied Sciences, Charotar University of Science & Technology, Changa, Gujarat 388 421, India; ^2^Department of Biotechnology, Ashok & Rita Patel Institute of Integrated Studies and Research in Biotechnology and Allied Sciences (ARIBAS), Sardar Patel University, New Vallabh Vidyanagar, Gujarat 388 121, India

## Abstract

Chemical synthesis of indigo poses harsh environmental hazards and adverse human health effects. This necessitates an environment-friendly and producer-friendly approach for indigo production. The present study was thus significant as it reports an indigenously isolated potential indigo pigment producing culture identified as *Pseudomonas* sp. HAV-1 with noteworthy antioxidant property. The bioindigo pigment was characterized using various analytical techniques. The pigment production was enhanced from 412 *μ*g mL^−1^ to 700 *μ*g mL^−1^ by optimizing the growth parameters. Furthermore, the antioxidant property of indigo pigment is hitherto unexplored. This property can significantly append to its therapeutic potential. The bioindigo pigment produced by *Pseudomonas* sp. HAV-1 depicted 2.2 *μ*M ascorbic acid equivalent antioxidant property. More to the point, the present work addresses a footstep towards green production of indigo.

## 1. Introduction

Indigoid pigments, a group of commodity chemicals, were anciently extracted from plants of* Indigofera* sp. Indigo (C. I. 73,000), a type of indigoid pigment, is one of the world's largest-produced dyes and has persistent applications in the textile industry [1]. However, the chemical synthesis of indigo not only imposed health hazards to workers but also led to severe environmental pollution due to the use of toxic materials like aniline, nitrobenzene, and metal salts [2, 3]. Since the responsiveness and concern towards protection of the environment is gaining momentum, interest lies in identifying eco-friendly methods for the indigo synthesis. As a result, microbial synthesis of indigoid pigments is commendable over chemical synthesis [4].

Most of the aromatic hydrocarbon-degrading bacteria represent the ability to synthesize indigo dye [4–7]. Therefore, such processes render dual advantage of removal of the recalcitrant aromatic hydrocarbons from the environment as well as bioindigo production. Several microbial processes for indigo synthesis have been reported, but the main apprehension is the lower productivity when compared to the chemical processes [8]. Hence, interest lies in isolation of microbes that can produce indigo at a substantial rate. Once potential indigo producing microorganisms have been identified, the rate of indigo synthesis can be augmented employing well designed optimization strategy. The present work employed optimization studies based on response surface method (RSM) theory, which studies the influence of identified parameters and their individual and interactive effects, by performing a series of planned experiments and analyzing the response statistically. Furthermore, indigoid pigments have been reported to possess antileukemic, hemostatic, antipyretic, anti-inflammatory, and sedative properties [9–12]. These are aromatic compounds that portray tremendous therapeutic potential in a number of diseases which includes Alzheimer's disease, certain forms of cancer, and delayed hypersensitivity [4]. The antioxidant property of indigo from plant source is scarcely reported [13] and reports are lacking on evaluation of antioxidant property of bacterial derived indigo. This property can considerably augment its therapeutic prospective.

With this background, the present study was aimed at isolation of indigo producing microorganisms with higher yield. Optimization study based on response surface method (RSM) was employed, which studies the influence of identified parameters along with their interactive effects, by performing a series of planned experiments and analyzing the response statistically. Antioxidant property of the bioindigo was explored to enhance its therapeutic potential.

## 2. Materials and Methods

### 2.1. Sample Collection, Enrichment, and Isolation of Indigo Producing Organisms

Soil samples were collected from oil contaminated sites of Vadodara, India, and were employed for isolation of naphthalene degrading organisms by enrichment technique using Bushnell Haas Broth (BHB) spiked with naphthalene (100 mg L^−1^) as a sole carbon source. The medium was inoculated with soil sample (10% w/v) and incubated under shaking condition (150 rpm at 30°C). Repetitive transfers were carried out into fresh naphthalene spiked medium until consistent visual growth and naphthalene degradation were observed. This enrichment culture was plated on Bushnell Haas agar containing naphthalene (100 mg L^−1^) and incubated at 30°C for 24 h. The isolates obtained upon enrichment were subjected to indigo biotransformation using indole (3 mM) as substrate. The isolate HAV-1 showed highest indigo formation was selected for further studies (refer to Figure  1 in Supplementary Material available online at http://dx.doi.org/10.1155/2014/109249). The isolate HAV-1 was routinely grown at 30°C in Bushnell Haas broth (BHB) with naphthalene (100 mg L^−1^) and incubated under shaking conditions for 24 h. This was used as inoculum in the present study unless specified.

### 2.2. Identification of Indigo Producing Culture HAV-1

The isolate HAV-1 was subjected to genomic DNA extraction by phenol-chloroform method as described by Ausubel et al., 1997 [14]. Universal bacterial 16S rRNA gene primers corresponding to* Escherichia coli* position 8f and 1492r were used for polymerase chain reaction (PCR) amplification of the 16S rRNA gene [15]. Purified PCR products were sequenced using internal overlapping primers [15]. Sequence was initially analyzed at NCBI server (http://www.ncbi.nlm.nih.gov/) using BLAST (blastn) tool and corresponding sequences were downloaded. Similarly matrix was prepared using Dnadist program in PHYLIP analysis package [16] using Jukes Cantor corrections. Phylogenetic tree was constructed by neighbour-joining method using the MEGA package.

### 2.3. Biosynthesis, Extraction, and Characterization of Blue Pigment

Isolate HAV-1 was inoculated (10%) in BHB spiked with naphthalene (100 mg/L) and incubated for 24 h at 30°C under shaking conditions. The broth was filtered using Whatman filter paper 1 to remove residual naphthalene and indole was added to the filtrate at a final concentration of 3 mM. The blue pigment from the whole culture broth was extracted using equal volume of chloroform and it was evaporated to obtain dried powder. The dried pigment was dissolved in 10 mL of HPLC grade chloroform. The pigment was analyzed by thin-layer chromatography (TLC) and Fourier Transform Infrared (FTIR) spectroscopy (NICOLET 6700, Thermo Scientific, USA). TLC and FTIR were performed as described earlier [4]. The pigment was also scanned in the range of 200–800 nm using double beam UV-Visible spectrophotometer (Systronics 2203, India). A similar characterization study was performed with commercial indigo for comparison purpose.

### 2.4. Induction Profile for Indigo Formation

In order to study the induction profile for indigo formation, each culture flask was harvested at a regular time interval of 4 hours. The culture broth of harvested flask was filtered using Whatman filter paper and the filtrate containing cells was used to determine indigo formation by method of O'Connor and Hartmans (1998) [17]. The absorbance of the indigo pigment was determined at 610 nm. Flask containing BHB spiked with (100 mg L^−1^) naphthalene along with 3 mM indole without isolate HAV-1 was used as control.

### 2.5. Effect of Inoculum Size and Indole Concentration on Indigo Production Using Response Surface Method

Response surface method was used to evaluate the individual as well as the combined effect of indole concentration and inoculum size on indigo formation using the central composite design (CCD). The “Design-Expert” version 8.0, State-Ease Inc., Minneapolis, USA, was used for experimental design, regression, and graphical analysis of the data obtained [14, 18]. Indole concentration and inoculum size were studied at five different levels (SM Table  1). A set of 13 experiments was performed (SM Table  2). The minimum and maximum ranges of variables were used and the full experiment design with respect to their values was generated. The data obtained from RSM on pigment production was subjected to the analysis of variance (ANOVA) and the results of RSM were used to fit a second order polynomial equation:

(1) $$\begin{array}{c} Y=\beta_{0}+\beta_{1}A+\beta_{2}B+\beta_{1}\beta_{2}AB+\beta_{1}\beta_{1}A^{2}+\beta_{2}\beta_{2}B^{2}, \end{array}$$

where *Y* is response variable (dependent variable), *β*_0_ is intercept (constant), *β*_1_, *β*_2_ are linear coefficients, *β*_1_ *β*_2_ is interaction coefficient, *β*_1_ *β*_1_, *β*_2_ *β*_2_ are squared coefficients, and *A*, *B*, *AB*, *A* ^2^, and *B* ^2^ are level of independent variables.

### 2.6. Evaluation of Antioxidant Activity of Biosynthesized Indigo Pigment

The antioxidant activity of the extracted indigo pigment as well as the commercial indigo pigment was determined employing the FRAP assay [19] and the ABTS assay [20] to obtain ferric reducing as well as total antioxidant power of the pigment. The antioxidant power was estimated in terms of ascorbic acid equivalents.

## 3. Results and Discussion

### 3.1. Isolation and Screening of Indigo Producing Bacteria

Three naphthalene degrading enrichment cultures designated as HAV-1, HAV-2, and HAV-3 were obtained after enrichment of hydrocarbon contaminated sediment samples. These isolates were found to be capable of naphthalene degradation (observed visually for the disappearance of naphthalene flakes from BHB broth). Among the three enrichment cultures, HAV-1 depicted the ability to utilize naphthalene at a faster rate (refer to Figure  1 in Supplementary Mterial). Hence HAV-1 was selected for further studies.

### 3.2. 16S rRNA-Based Culture Identification

**Figure 1:**
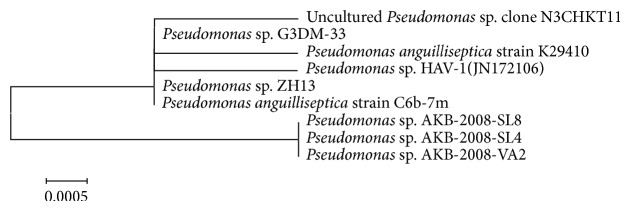
Phylogenetic tree derived from 16S rRNA gene sequence of isolate HAV-1. Sequences of closest phylogenetic neighbors obtained by NCBI BLAST (*n*) analysis; numbers in the parenthesis indicate accession numbers of corresponding sequences. The NJ-tree was constructed using neighbor joining algorithm with Kimura 2 parameter distances in MEGA 5.0 software.

Isolate HAV-1 was identified using 16S rRNA gene based approach. The DNA sequencing and BLAST analysis of 16S rRNA gene sequence of the isolate HAV-1 showed maximum sequence homology (99%) with the complete sequence of* Pseudomonas* sp. 16S rRNA gene. Phylogenetic analysis indicates that the newly isolated strain HAV-1 is affiliated to* Pseudomonas* species and hence in the present study it is referred to as* Pseudomonas* sp. HAV-1. The phylogeny cluster of HAV-1 along with related* Pseudomonas* species (a Gamma-proteobacteria) is depicted in Figure 1. The gene sequence was submitted to Genbank with accession number JN172106.

### 3.3. Characterization of Blue Pigment

**Figure 2:**
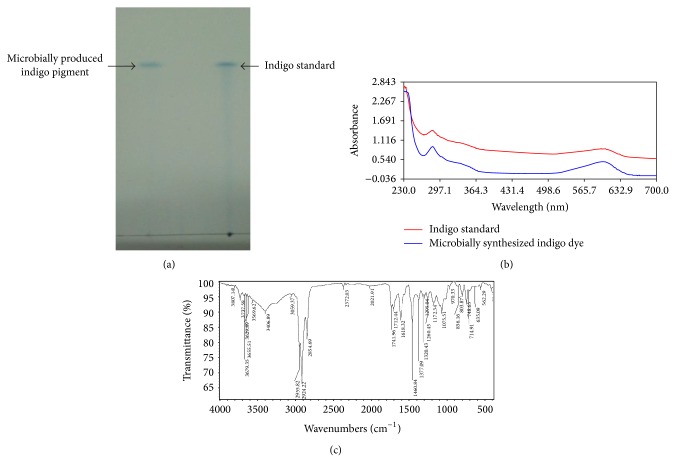
(a) TLC analysis of indigo pigment produced by* Pseudomonas* sp. HAV-1. (b) UV-Visible scan of indigo pigment produced by* Pseudomonas* sp. HAV-1. (c) FT-IR spectrum of indigo pigment produced by* Pseudomonas* sp. HAV-1.

The blue pigment produced by the isolate* Pseudomonas* sp. HAV-1 was characterized by TLC and FTIR. Indigo produced portrayed only one band when analyzed by TLC. The *R*_*f*_ value of indigo pigment matched exactly that of the commercial indigo pigment (Figure 2(a)). This implies that the blue pigment produced by isolate* Pseudomonas* sp. HAV-1 is indigo pigment. This was supported by the UV-Visible spectrum of the extracted blue pigment which was identical to that of the commercial indigo pigment (Figure 2(b)). The infrared spectrum accounted for the specificity of any compound and can be used as its “fingerprint” for identification purposes.  Figure 2(c) depicts the infrared spectrum of the extracted blue pigment. The FT-IR spectrum of the purified blue pigment shows a peak at 3,398 cm^−1^ that is attributed to the N–H stretching in secondary amines. A peak at 1,617 cm^−1^ may be attributed to C=O stretching coupled with C=C stretching. Further, weak bands in the range of 1,350–1,000 cm^−1^ have contributions from C–N stretching of the amine group. The peak around 735–770 cm^−1^ may be assigned to C–H out of the plane bending in aromatic group. Thus, the IR spectrum of the microbial synthesized compound supports the identification of blue pigment as indigo dye.

### 3.4. Time Course Study for Indigo Formation by HAV-1

**Figure 3:**
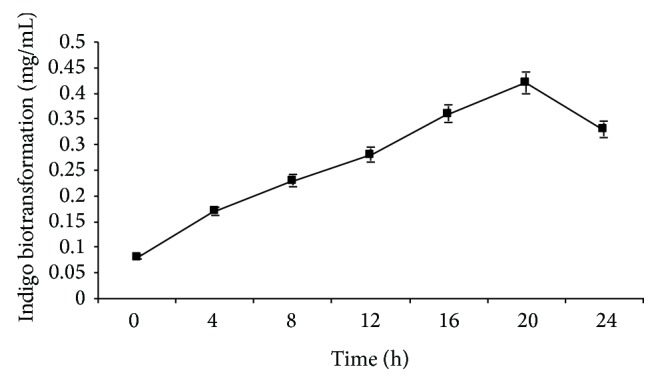
Induction profile of* Pseudomonas* sp. HAV-1 for indigo biotransformation using 3 mM indole concentration at 30°C with initial pH of 7.0.

Indigo formation increased linearly during initial 20 h of the growth and reached maximum 412 *μ*g mL^−1^ and decreased thereafter (Figure 3). No indigo formation was observed in the control flask suggesting the role of* Pseudomonas* sp. HAV-1 in indigo biosynthesis.

### 3.5. Effect of Inoculum Size and Indole Concentration on Indigo Formation

**Figure 4:**
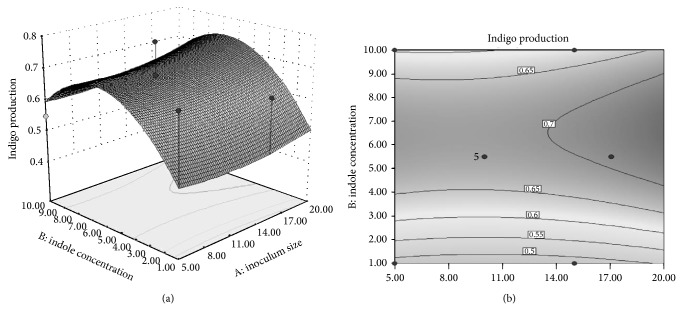
(a) 3D plot showing the interaction effect of indole concentration and inoculum size on indigo formation by* Pseudomonas* sp. HAV-1 and (b) Contour plot showing the interaction effect of indole concentration and inoculum size on indigo formation by* Pseudomonas* sp. HAV-1.

Response surface method was used to understand individual as well as combined effect of inoculum size and indole concentration. From analysis of variance (ANOVA) (Table 1) it was established that neither inoculum size nor indole concentration was significant (*P* values less than 0.05 indicate model terms are significant). Moreover, combined effect of these factors was not significant for indigo production. The contour plot in Figure 4 exhibits the behavior of indigo production with respect to changes in the initial inoculum size and indole concentration in the selected range. Indigo production was highest (700 *μ*g mL^−1^) by increasing inoculum size to 14% with indole concentration of 7 mM. Furthermore, the indigo formation by* Pseudomonas* sp. HAV-1 was superior when compared to other reported studies for indigo production (Table 2) [21, 22]. This implies that* Pseudomonas* sp. HAV-1 possesses tremendous potential as candidate for commercial production of indigo pigment.

### 3.6. Antioxidant Activity

The antioxidant activity of the microbially synthesized indigo pigment as determined by FRAP and ABTS assay was found to be comparable with the commercial indigo pigment (Table 3). To the best of our knowledge, this is the first report depicting the antioxidant activity of the microbially produced indigo pigment.

## 4. Conclusion

The strain* Pseudomonas* sp. HAV-1 exhibits exceptional potential for bioindigo production (700 *μ*g mL^−1^) with 20 hours' induction time. Moreover, this study is the first account on exploration of antioxidant activity of bioindigo pigment. The present study is, besides, a move towards green synthesis of indigo dye.

## Supplementary Material

Figure 1 contains the information about the comparatives of indigo production by three different isolates which formed the basis for selection of the isolate HAV-1 for further studies. Table 1 and table 2 respectively depict the range of values chosen for variable parameters for the response surface method and the coded as well as the actual experimental design to execute statistical optimization.

## Figures and Tables

**Table 1:** ANOVA for response surface quadratic model.

Source	Sum of squares	df	Mean square	*F* value	*P* value	
					Prob > *F*	
Model	0.178603	3	0.059534	10.7657	0.0219	Significant
*A*: inoculum size	0.000182	1	0.000182	0.00634	0.9388	
*B*: indole concentration	0.033406	1	0.033406	1.164996	0.3162	
*AB*	0.000156	1	0.000156	0.005449	0.9432	
*A* ^2^	0.000636	1	0.000636	0.022184	0.8858	
*B* ^2^	0.157436	1	0.157436	5.490418	0.0516	
Residual	0.200723	7	0.028675			
Lack of fit	0.197208	5	0.039442	1.375483	0.3378	Not significant

*R* ^2^: 0.93, Adj. *R* ^2^: 0.85, Pred. *R* ^2^: 0.97, Adqt. Prec.: 15.

**Table 2:** Indigo production by different bacterial cultures.

Indigo produced (*µ*g mL^−1^)	Time (h)	Culture	Reference
700^*^	24	*Pseudomonas* sp. HAV-1	Present study
292	24	*Acinetobacter *ST-550	[1]
662	24	*E. coli *	[21]
246	8	*Pseudomonas *sp. HOB1	[4]
30	24	*Comamonas *sp.	[22]

^*^After optimization.

**Table 3:** Antioxidant property of extracted indigo as well as commercial indigo.

Assay	Antioxidant property of commercial indigo	Antioxidant property of indigo produced by *Pseudomonas* sp. HAV-1
FRAP	0.13 mM FeSO_4_ equivalent	0.50 mM FeSO_4_ equivalent
ABTS	1 *μ*M ascorbic acid equivalent	2.2 *μ*M ascorbic acid equivalent
