# Supplementary material for: Biotransformation of Indigo Pigment by Indigenously Isolated Pseudomonas sp. HAV-1 and Assessment of Its Antioxidant Property
Source: Biotechnol Res Int. 2014 Nov 17;2014:109249. doi: 10.1155/2014/109249 (PMC4251628; doi:10.1155/2014/109249)
Supplement: Supplementary file 1 — Figure 1 contains the information about the comparatives of indigo production by three different isolates which formed the basis for selection of the isolate HAV-1 for further studies. Table 1 and table 2 respectively depict the range of values chosen for variable parameters for the response surface method and the coded as well as the actual experimental design to execute statistical optimization. [file 109249.f1.pdf]

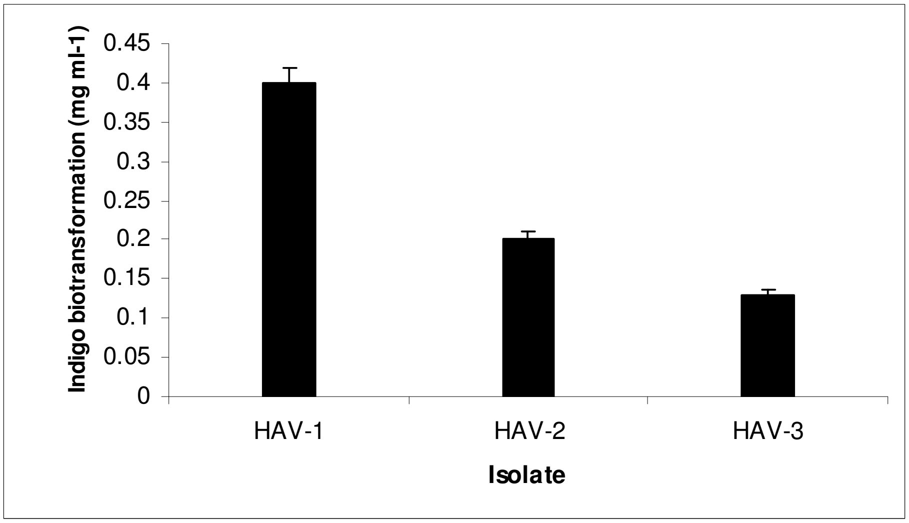

Figure 1: Screening of indigo producing bacterial isolates (HAV-1, HAV-2 and HAV-3) using 3 mM indole concentration at 30°C with initial pH of 7.0

Table 1: Range of values for the response surface method

Independent variables	Levels				
	-α	-1	0	+1	+α
Inoculum size (%)	-2.9	5	10	15	17.7
Indole mM	-0.86	1	5.5	10	11.86

Table 2: Experimental design (CCD) in terms of actual and coded value

Run	A: Inoculum size (%)		B: Indole concentration mM	
	Actual	Coded	Actual	Coded
1.	5	-1	1	-1
2.	15	1	1	-1
3.	5	-1	10	1
4.	15	1	10	1
5.	2.92	-1.41	5.5	0
6.	17.07	1.41	5.5	0
7.	10	0	-0.86	-1.41
8.	10	0	11.8	1.41
9.	10	0	5.5	0
10.	10	0	5.5	0
11.	10	0	5.5	0
12.	10	0	5.5	0
13.	10	0	5.5	0
